# 3-{3,5-Bis[(2-but­oxy­eth­oxy)carbon­yl]-2,6-dimethyl-1,4-dihydro­pyridin-4-yl}-1-[(3,4,5-trimeth­oxy­benzo­yl)meth­yl]pyridinium bromide

**DOI:** 10.1107/S1600536812049896

**Published:** 2012-12-08

**Authors:** Imants Bisenieks, Anatoly Mishnev, Imanta Bruvere, Brigita Vigante

**Affiliations:** a Latvian Institute of Organic Synthesis, 21 Aizkraukles Street, Riga LV-1006, Latvia

## Abstract

In the title salt, C_37_H_51_N_2_O_10_^+^·Br^−^, the 1,4-dihydro­pyridine (1,4-DHP) ring adopts a slighly puckered boat conformation. The N and opposite C atoms deviate from the least-squares plane calculated through the four other ring atoms by 0.068 (5) and 0.224 (5) Å, respectively. The orientation of both C=O groups is similar (*cis* with respect to the double bonds of 1,4-DHP. The pyridinium ring has an axial orientation with respect to the1,4-DHP ring and is almost perpendicular to the least-squares plane of the 1,4-DHP ring, making a dihedral angle of 89.2 (3)°. The mol­ecule has a compact shape due to the parallel orientation of the long-chain substituents. One of the but­oxy groups was fond to be disordered (occupancy ratio 0.70:0.30). In the crystal, the bromide anion accepts a weak hydrogen bond from the N—H group of a neighboring 1,4-DHP ring.

## Related literature

For general information on the relationship between 1,4-dihydro­pyridine ring substituents and pharmaceutical effects, see: Hasko & Pacher (2008 ▶); Niebauer & Robinson (2006 ▶); Ruiz *et al.* (2012 ▶); Swarnalatha *et al.* (2011 ▶). For the synthesis of the DHP 3-pyridyl derivative, see: Saini *et al.* (2008 ▶).
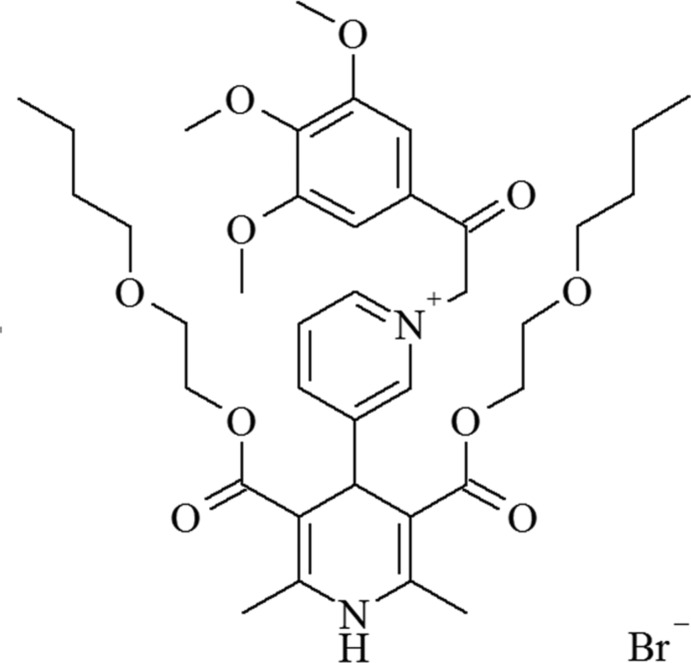


## Experimental

### 

#### Crystal data

C_37_H_51_N_2_O_10_^+^·Br^−^
*M* *_r_* = 763.71
Triclinic, 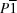

*a* = 8.9501 (2) Å
*b* = 12.4741 (3) Å
*c* = 17.6994 (5) Å
α = 93.057 (1)°
β = 91.658 (1)°
γ = 108.024 (1)°
*V* = 1874.25 (8) Å^3^
*Z* = 2
Mo *K*α radiation
μ = 1.16 mm^−1^
*T* = 190 K
0.32 × 0.18 × 0.16 mm

#### Data collection

Nonius KappaCCD diffractometer
13618 measured reflections
8822 independent reflections
6509 reflections with *I* > 2σ(*I*)
*R*_int_ = 0.035

#### Refinement

*R*[*F* ^2^ > 2σ(*F* ^2^)] = 0.056
*wR*(*F* ^2^) = 0.127
*S* = 1.03
8822 reflections
469 parameters
4 restraints
H-atom parameters constrained
Δρ_max_ = 1.15 e Å^−3^
Δρ_min_ = −0.41 e Å^−3^

### 

Data collection: *COLLECT* (Hooft, 1998 ▶); cell refinement: *SCALEPACK* (Otwinowski & Minor, 1997 ▶); data reduction: *DENZO* (Otwinowski & Minor, 1997 ▶) and *SCALEPACK*; program(s) used to solve structure: *SHELXS97* (Sheldrick, 2008 ▶); program(s) used to refine structure: *SHELXL97* (Sheldrick, 2008 ▶); molecular graphics: *ORTEP-3* (Farrugia, 2012 ▶); software used to prepare material for publication: *SHELXL97*.

## Supplementary Material

Crystal structure: contains datablock(s) I, global. DOI: 10.1107/S1600536812049896/vm2184sup1.cif

Structure factors: contains datablock(s) I. DOI: 10.1107/S1600536812049896/vm2184Isup2.hkl

Supplementary material file. DOI: 10.1107/S1600536812049896/vm2184Isup3.cml

Additional supplementary materials:  crystallographic information; 3D view; checkCIF report

## Figures and Tables

**Table 1:** Hydrogen-bond geometry (Å, °)

*D*—H⋯*A*	*D*—H	H⋯*A*	*D*⋯*A*	*D*—H⋯*A*
N1—H1⋯Br1^i^	0.86	2.61	3.421 (2)	157

Symmetry code: (i) 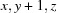.

**Fig. 1.**
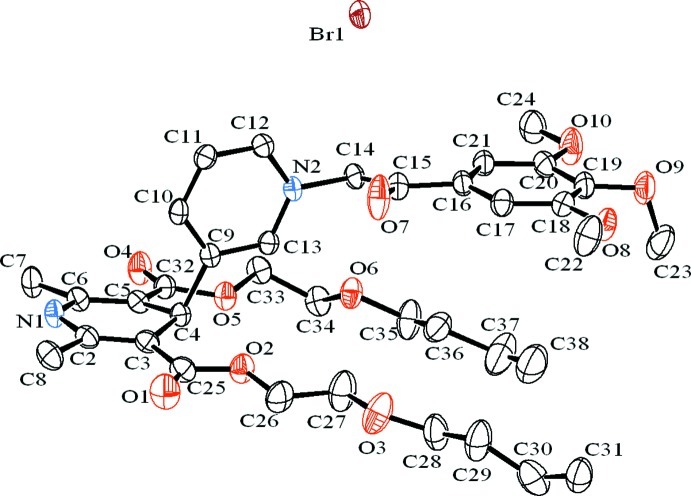
The molecular structure of the title compound showing 50% probability elipsoids. No hydrogen atoms shown for clarity. For the disordered butoxy fragment only atoms with the higher occupation factor are shown (C28 - C31).

## supplementary crystallographic information

### Comment

Nowadays considerable attention is paid to the synthesis of 1,4-dihydropyridine (1,4-DHP) derivatives because of their wide spectrum of biological activity. Slight variations in 1,4-DHP ring substituents can result in considerable changes in pharmacological effects (Swarnalatha *et al.*, 2011). 1,4-Dihydropyridines are considered as privileged structures, because these compounds are capable to bind to multiple receptors with high affinity (Ruiz *et al.*, 2012).

It was found that the concentration of adenosine, the natural ligand of the A_2A_ receptor, changes in the ischemia, hypoxia and inflammation conditions (Hasko & Pacher, 2008). The A_2A_ receptor is believed to play a role in cardioprotection, inflammation, stroke and certain central nervous system disorders (Niebauer & Robinson, 2006). We were looking for molecules based on 1,4-DHP able to bind A_2A_ adenosine receptors and possessing enhanced water solubility.

Fig. 1 shows a view of the crystal structure of the title compound. For the disordered butoxy fragment only atoms with the higher occupation factor are shown. In the crystal structure, the 1,4-DHP ring adopts a slightly puckered boat conformation. Atoms N1 and C4 deviate from the least-squares plane calculated through the four other ring atoms by 0.068 (5) Å and 0.224 (5) Å, respectively. The orientation of both C=O groups is *cis* with respect to the double bonds of 1,4-DHP. The pyridinium ring has an axial orientation with respect to the 1,4-DHP ring and is almost perpendicular to the least-squares average plane of the 1,4-DHP ring with a dihedral angle between both planes of 89.2 (3)°. The molecule has a compact shape with all long chain substituents oriented approximately in one direction. All bonds in the substituents at the 3 and 5 position of 1,4-DHP have *trans* orientation except for bonds C29B—C30B and C33—C34 (gau*che*^-^). The bromine anion forms a weak hydrogen bond with N1—H1 of a neighboring 1,4-DHP ring. The distance between the bromine ion and the positively charged N2 atom is 4.185 (5) Å.

### Experimental

First the DHP 3-pyridyl derivative was obtained by means of the Hantzsch method as described by Saini *et al.* (2008). After the structure confirmation the intermediate (1 mmol) was added to α-bromo-(3,4,5-trimethoxy)-acetophenone (1 mmol) in 30 ml acetone. Reaction mixture was boiled for 24 h, and after completion (monitored by TLC) cooled to ambient temperature. This procedure gives the title compound pyridinium salt as block crystals, suitable for X-ray analysis. ^1^H-NMR (400 MHz, DMSO-*d_6_*), *δ*/p.p.m.: 9.08 (m, 1H, py-2-H), 8.78–8.82 (m, 1H, py-6-H), 8.46 (d, 1H, *J*=8.0 Hz, py-4-H), 7.71–7.77(m, 2H, py-5-H and N—H), 7.46 (s, 2H, Ph-2,6-H), 6.95 (m, 2H, N^+^CH_2_), 5.16(s, 1H, 4-H), 4.17 (t, 4H, *J*=4.8 Hz, COOCH_2_CH_2_), 3.98 (s, 6H,Ar—H), 3.92 (s, 3H, Ar-4-OCH_3_), 3.52–3.62 (m, 4H, 3,5-COOCH_2_CH_2_),3.36–3.45 (m, 4H, 3,5-CH_2_CH_2_CH_2_CH_3_), 2.46 (s, 6H, 2,6-CH_3_),1.46–1.53(m, 4H, 3,5-CH_2_CH_2_CH_2_CH_3_), 1.25–1,35 (m, 4H,3,5-CH_2_CH_2_CH_2_CH_3_), 0.85–0.89 (t, 6H, *J*=7.6 Hz,3,5-CH_2_CH_2_CH_2_CH_3_); MS (ESI) m/*z*: 684 [M—Br]^+^; Anal. Calcd for C_37_H_51_BrN_2_O_10_: C, 58.19; H, 6.73; N, 3.67; found: C, 58.11; H, 6.76; N, 3.61.

### Refinement

The H-atoms were included in the refinement at calculated positions (N—H = 0.86 Å, C—H = 0.93 to 0.98 Å) and treated using a riding-model approximation as implemented in *SHELXL97* software. Disorder was detected in the butoxy group with occupancies of 0.7 for atoms C28, C29, C30 and C31 and 0.3 for C28B, C29B, C30B and C31B. The maximum difference density is rather high (1.15 e Å^-3^) because of Fourier series truncation errors expected for a structure containing a heavy atom Br.

### Crystal data

**Table:**

C_37_H_51_N_2_O_10_^+^·Br^−^	*Z* = 2
*M**_r_* = 763.71	*F*(000) = 804
Triclinic, *P*1	*D*_x_ = 1.353 Mg m^−^^3^
Hall symbol: -P 1	Mo *K*α radiation, λ = 0.71073 Å
*a* = 8.9501 (2) Å	Cell parameters from 20441 reflections
*b* = 12.4741 (3) Å	θ = 1.0–27.9°
*c* = 17.6994 (5) Å	µ = 1.16 mm^−^^1^
α = 93.057 (1)°	*T* = 190 K
β = 91.658 (1)°	Block, colourless
γ = 108.024 (1)°	0.32 × 0.18 × 0.16 mm
*V* = 1874.25 (8) Å^3^	

### Data collection

**Table:**

Nonius KappaCCD diffractometer	6509 reflections with *I* > 2σ(*I*)
Radiation source: fine-focus sealed tube	*R*_int_ = 0.035
Graphite monochromator	θ_max_ = 27.8°, θ_min_ = 2.1°
CCD scans	*h* = −11→11
13618 measured reflections	*k* = −16→16
8822 independent reflections	*l* = −23→23

### Refinement

**Table:**

Refinement on *F*^2^	Primary atom site location: structure-invariant direct methods
Least-squares matrix: full	Secondary atom site location: difference Fourier map
*R*[*F*^2^ > 2σ(*F*^2^)] = 0.056	Hydrogen site location: inferred from neighbouring sites
*wR*(*F*^2^) = 0.127	H-atom parameters constrained
*S* = 1.03	*w* = 1/[σ^2^(*F*_o_^2^) + (0.040*P*)^2^ + 2.*P*] where *P* = (*F*_o_^2^ + 2*F*_c_^2^)/3
8822 reflections	(Δ/σ)_max_ = 0.001
469 parameters	Δρ_max_ = 1.15 e Å^−^^3^
4 restraints	Δρ_min_ = −0.41 e Å^−^^3^

### Special details

**Table:**

Geometry. All e.s.d.'s (except the e.s.d. in the dihedral angle between two l.s. planes) are estimated using the full covariance matrix. The cell e.s.d.'s are taken into account individually in the estimation of e.s.d.'s in distances, angles and torsion angles; correlations between e.s.d.'s in cell parameters are only used when they are defined by crystal symmetry. An approximate (isotropic) treatment of cell e.s.d.'s is used for estimating e.s.d.'s involving l.s. planes.
Refinement. Refinement of *F*^2^ against ALL reflections. The weighted *R*-factor *wR* and goodness of fit *S* are based on *F*^2^, conventional *R*-factors *R* are based on *F*, with *F* set to zero for negative *F*^2^. The threshold expression of *F*^2^ > σ(*F*^2^) is used only for calculating *R*-factors(gt) *etc*. and is not relevant to the choice of reflections for refinement. *R*-factors based on *F*^2^ are statistically about twice as large as those based on *F*, and *R*- factors based on ALL data will be even larger.

### Fractional atomic coordinates and isotropic or equivalent isotropic displacement parameters (Å^2^)

**Table:**

	*x*	*y*	*z*	*U*_iso_*/*U*_eq_	Occ. (<1)
Br1	0.37986 (4)	0.16649 (2)	0.524390 (18)	0.03630 (11)	
O5	−0.0812 (2)	0.54844 (16)	0.61743 (11)	0.0294 (4)	
O6	−0.1497 (2)	0.35179 (17)	0.69842 (12)	0.0354 (5)	
N2	0.2738 (3)	0.43048 (18)	0.64368 (12)	0.0233 (5)	
O8	0.2588 (3)	0.03534 (18)	0.93630 (12)	0.0410 (5)	
O4	−0.1071 (2)	0.62698 (18)	0.50837 (12)	0.0377 (5)	
O10	−0.0811 (3)	−0.10148 (19)	0.72532 (13)	0.0512 (6)	
O2	0.2910 (3)	0.71278 (18)	0.81572 (11)	0.0362 (5)	
N1	0.2834 (3)	0.90617 (19)	0.59677 (14)	0.0310 (5)	
H1	0.3253	0.9628	0.5704	0.037*	
O9	0.0265 (3)	−0.12535 (18)	0.86279 (13)	0.0474 (6)	
O1	0.4524 (3)	0.8906 (2)	0.82333 (13)	0.0491 (6)	
C32	−0.0378 (3)	0.6335 (2)	0.56916 (15)	0.0264 (6)	
O7	0.4044 (3)	0.3582 (2)	0.75908 (14)	0.0528 (7)	
C10	0.4047 (3)	0.6509 (2)	0.61258 (15)	0.0263 (6)	
H10	0.4495	0.7258	0.6015	0.032*	
C13	0.2062 (3)	0.5076 (2)	0.66831 (15)	0.0243 (6)	
H13	0.1170	0.4854	0.6965	0.029*	
C4	0.1812 (3)	0.7049 (2)	0.67322 (15)	0.0243 (6)	
H4	0.1025	0.6725	0.7099	0.029*	
C5	0.0976 (3)	0.7251 (2)	0.60136 (15)	0.0248 (6)	
C14	0.2046 (3)	0.3118 (2)	0.66100 (16)	0.0264 (6)	
H14A	0.2135	0.2628	0.6181	0.032*	
H14B	0.0939	0.2967	0.6701	0.032*	
C6	0.1559 (3)	0.8212 (2)	0.56498 (16)	0.0279 (6)	
C16	0.2225 (3)	0.1752 (2)	0.76203 (16)	0.0285 (6)	
C12	0.4066 (3)	0.4596 (2)	0.60494 (15)	0.0261 (6)	
H12	0.4516	0.4050	0.5887	0.031*	
C11	0.4746 (3)	0.5706 (2)	0.58965 (16)	0.0277 (6)	
H11	0.5674	0.5918	0.5640	0.033*	
C15	0.2887 (3)	0.2872 (2)	0.73052 (17)	0.0309 (6)	
C9	0.2681 (3)	0.6197 (2)	0.65203 (14)	0.0229 (5)	
C33	−0.1872 (3)	0.4421 (2)	0.58642 (17)	0.0312 (6)	
H33A	−0.1295	0.4009	0.5576	0.037*	
H33B	−0.2663	0.4550	0.5527	0.037*	
C2	0.3479 (3)	0.9060 (2)	0.66820 (16)	0.0283 (6)	
C21	0.1014 (3)	0.0893 (2)	0.72309 (17)	0.0304 (6)	
H21	0.0643	0.0996	0.6753	0.036*	
O3	0.3297 (3)	0.5876 (3)	0.98920 (14)	0.0642 (8)	
C17	0.2812 (3)	0.1598 (2)	0.83288 (17)	0.0310 (6)	
H17	0.3636	0.2168	0.8576	0.037*	
C34	−0.2639 (3)	0.3750 (3)	0.64977 (18)	0.0346 (7)	
H34A	−0.3208	0.4167	0.6785	0.041*	
H34B	−0.3389	0.3045	0.6294	0.041*	
C7	0.0949 (4)	0.8480 (3)	0.49096 (17)	0.0372 (7)	
H7A	0.1144	0.7999	0.4509	0.056*	
H7B	0.1474	0.9255	0.4818	0.056*	
H7C	−0.0162	0.8359	0.4928	0.056*	
C19	0.0922 (4)	−0.0265 (2)	0.82862 (17)	0.0358 (7)	
C25	0.3563 (4)	0.8138 (3)	0.78638 (17)	0.0326 (6)	
C20	0.0376 (4)	−0.0115 (2)	0.75696 (17)	0.0352 (7)	
C3	0.2964 (3)	0.8140 (2)	0.70859 (15)	0.0269 (6)	
C8	0.4740 (4)	1.0140 (2)	0.69322 (19)	0.0386 (7)	
H8A	0.4357	1.0542	0.7319	0.058*	
H8B	0.5024	1.0598	0.6508	0.058*	
H8C	0.5647	0.9972	0.7129	0.058*	
C26	0.3417 (4)	0.7075 (3)	0.89303 (17)	0.0438 (8)	
H26A	0.3135	0.7627	0.9255	0.053*	
H26B	0.4550	0.7240	0.8971	0.053*	
C18	0.2157 (4)	0.0586 (3)	0.86639 (16)	0.0323 (6)	
C24	−0.1484 (4)	−0.0902 (3)	0.65342 (19)	0.0461 (8)	
H24A	−0.1965	−0.0314	0.6577	0.069*	
H24B	−0.2264	−0.1602	0.6366	0.069*	
H24C	−0.0675	−0.0715	0.6174	0.069*	
C36	−0.1104 (4)	0.2827 (3)	0.81904 (19)	0.0467 (9)	
H36A	−0.0652	0.3591	0.8414	0.056*	
H36B	−0.0256	0.2582	0.7991	0.056*	
C22	0.3969 (4)	0.1114 (3)	0.9727 (2)	0.0550 (10)	
H22A	0.4852	0.1150	0.9423	0.083*	
H22B	0.4143	0.0857	1.0213	0.083*	
H22C	0.3849	0.1852	0.9791	0.083*	
C23	−0.1099 (5)	−0.1226 (3)	0.9026 (2)	0.0635 (12)	
H23A	−0.1531	−0.1927	0.9258	0.095*	
H23B	−0.1872	−0.1118	0.8676	0.095*	
H23C	−0.0808	−0.0615	0.9409	0.095*	
C35	−0.2229 (4)	0.2823 (3)	0.7565 (2)	0.0519 (9)	
H32A	−0.2704	0.2055	0.7352	0.062*	
H32B	−0.3060	0.3089	0.7763	0.062*	
C27	0.2631 (5)	0.5922 (3)	0.9163 (2)	0.0582 (10)	
H27A	0.1505	0.5784	0.9182	0.070*	
H27B	0.2819	0.5362	0.8810	0.070*	
C37	−0.1891 (6)	0.2048 (5)	0.8804 (3)	0.0757 (13)	
H37A	−0.2754	0.2289	0.8989	0.091*	
H37B	−0.2336	0.1288	0.8574	0.091*	
C38	−0.0868 (6)	0.2013 (5)	0.9449 (3)	0.0794 (14)	
H38A	−0.1465	0.1517	0.9804	0.119*	
H38B	−0.0429	0.2759	0.9688	0.119*	
H38C	−0.0035	0.1741	0.9279	0.119*	
C28	0.2319 (8)	0.5017 (5)	1.0321 (3)	0.0560 (15)	0.70
H28A	0.1895	0.4332	0.9995	0.067*	0.70
H28B	0.1442	0.5254	1.0490	0.067*	0.70
C29	0.3162 (9)	0.4762 (6)	1.0992 (3)	0.0622 (18)	0.70
H29A	0.3861	0.5469	1.1224	0.075*	0.70
H29B	0.3811	0.4313	1.0818	0.075*	0.70
C30	0.2131 (9)	0.4145 (6)	1.1593 (4)	0.0618 (18)	0.70
H30A	0.1629	0.4647	1.1840	0.074*	0.70
H30B	0.1310	0.3505	1.1353	0.074*	0.70
C31	0.3027 (7)	0.3732 (4)	1.2181 (3)	0.0540 (14)	0.70
H31A	0.2320	0.3353	1.2550	0.081*	0.70
H31B	0.3832	0.4363	1.2426	0.081*	0.70
H31C	0.3501	0.3217	1.1942	0.081*	0.70
C28B	0.300 (2)	0.4835 (14)	1.0220 (10)	0.090*	0.30
H28C	0.3490	0.4347	0.9949	0.108*	0.30
H28D	0.1879	0.4450	1.0234	0.108*	0.30
C29B	0.376 (2)	0.5201 (13)	1.1025 (9)	0.062 (5)*	0.30
H29C	0.4893	0.5497	1.0998	0.074*	0.30
H29D	0.3390	0.5795	1.1247	0.074*	0.30
C30B	0.334 (2)	0.4200 (13)	1.1518 (9)	0.071 (4)*	0.30
H30C	0.3348	0.3528	1.1218	0.086*	0.30
H30D	0.4134	0.4333	1.1927	0.086*	0.30
C31B	0.177 (3)	0.400 (3)	1.1845 (16)	0.100*	0.30
H31D	0.1543	0.3340	1.2134	0.150*	0.30
H31E	0.0983	0.3877	1.1443	0.150*	0.30
H31F	0.1777	0.4640	1.2168	0.150*	0.30

### Atomic displacement parameters (Å^2^)

**Table:**

	*U*^11^	*U*^22^	*U*^33^	*U*^12^	*U*^13^	*U*^23^
Br1	0.0452 (2)	0.02315 (15)	0.03853 (18)	0.00647 (12)	0.00596 (13)	0.00699 (11)
O5	0.0275 (10)	0.0252 (10)	0.0299 (10)	0.0006 (8)	−0.0061 (8)	0.0031 (8)
O6	0.0267 (11)	0.0348 (12)	0.0421 (12)	0.0041 (9)	0.0009 (9)	0.0138 (9)
N2	0.0235 (12)	0.0192 (11)	0.0251 (11)	0.0036 (9)	−0.0029 (9)	0.0043 (9)
O8	0.0500 (14)	0.0376 (12)	0.0297 (11)	0.0037 (10)	−0.0017 (10)	0.0126 (9)
O4	0.0413 (12)	0.0334 (11)	0.0328 (11)	0.0039 (10)	−0.0123 (9)	0.0072 (9)
O10	0.0711 (17)	0.0278 (12)	0.0394 (13)	−0.0068 (11)	−0.0079 (12)	0.0053 (10)
O2	0.0437 (12)	0.0343 (12)	0.0262 (11)	0.0063 (10)	−0.0071 (9)	0.0024 (9)
N1	0.0343 (13)	0.0214 (12)	0.0345 (13)	0.0036 (10)	0.0003 (11)	0.0076 (10)
O9	0.0687 (16)	0.0267 (11)	0.0385 (13)	0.0011 (11)	0.0061 (11)	0.0095 (9)
O1	0.0562 (15)	0.0393 (13)	0.0401 (13)	0.0007 (11)	−0.0164 (11)	−0.0043 (10)
C32	0.0262 (14)	0.0264 (14)	0.0263 (14)	0.0077 (12)	−0.0006 (11)	0.0030 (11)
O7	0.0429 (14)	0.0422 (14)	0.0581 (15)	−0.0104 (11)	−0.0206 (12)	0.0251 (11)
C10	0.0277 (14)	0.0211 (13)	0.0261 (14)	0.0013 (11)	−0.0014 (11)	0.0052 (11)
C13	0.0237 (14)	0.0246 (14)	0.0236 (13)	0.0061 (11)	−0.0013 (11)	0.0036 (11)
C4	0.0235 (14)	0.0223 (13)	0.0260 (14)	0.0056 (11)	−0.0010 (11)	0.0029 (11)
C5	0.0246 (14)	0.0219 (13)	0.0276 (14)	0.0075 (11)	−0.0016 (11)	0.0001 (11)
C14	0.0238 (14)	0.0185 (13)	0.0336 (15)	0.0017 (11)	−0.0001 (11)	0.0035 (11)
C6	0.0304 (15)	0.0246 (14)	0.0299 (15)	0.0102 (12)	−0.0010 (12)	0.0027 (11)
C16	0.0279 (15)	0.0243 (14)	0.0352 (16)	0.0092 (12)	0.0052 (12)	0.0095 (12)
C12	0.0248 (14)	0.0252 (14)	0.0283 (14)	0.0079 (11)	−0.0007 (11)	0.0022 (11)
C11	0.0231 (14)	0.0285 (14)	0.0291 (15)	0.0043 (11)	0.0024 (11)	0.0044 (11)
C15	0.0285 (15)	0.0290 (15)	0.0346 (16)	0.0073 (13)	0.0007 (12)	0.0083 (12)
C9	0.0252 (14)	0.0200 (13)	0.0210 (13)	0.0041 (11)	−0.0066 (10)	0.0007 (10)
C33	0.0272 (15)	0.0258 (14)	0.0356 (16)	0.0022 (12)	−0.0060 (12)	−0.0013 (12)
C2	0.0287 (15)	0.0223 (14)	0.0327 (15)	0.0069 (12)	0.0006 (12)	−0.0016 (11)
C21	0.0352 (16)	0.0246 (14)	0.0303 (15)	0.0069 (12)	0.0029 (12)	0.0056 (11)
O3	0.0659 (18)	0.082 (2)	0.0400 (14)	0.0141 (16)	−0.0042 (12)	0.0262 (14)
C17	0.0261 (15)	0.0301 (15)	0.0351 (16)	0.0053 (12)	0.0011 (12)	0.0072 (12)
C34	0.0265 (15)	0.0303 (16)	0.0421 (18)	0.0024 (12)	−0.0052 (13)	0.0029 (13)
C7	0.0472 (19)	0.0268 (15)	0.0349 (17)	0.0071 (14)	−0.0043 (14)	0.0089 (13)
C19	0.0495 (19)	0.0229 (14)	0.0328 (16)	0.0068 (14)	0.0066 (14)	0.0066 (12)
C25	0.0335 (16)	0.0304 (15)	0.0329 (16)	0.0097 (13)	−0.0038 (13)	−0.0024 (13)
C20	0.0442 (18)	0.0212 (14)	0.0363 (17)	0.0048 (13)	0.0032 (14)	0.0005 (12)
C3	0.0276 (14)	0.0246 (14)	0.0272 (14)	0.0072 (12)	−0.0023 (11)	−0.0030 (11)
C8	0.0358 (17)	0.0247 (15)	0.0508 (19)	0.0040 (13)	−0.0024 (14)	−0.0016 (14)
C26	0.054 (2)	0.050 (2)	0.0262 (16)	0.0135 (17)	−0.0073 (14)	0.0052 (14)
C18	0.0379 (17)	0.0318 (16)	0.0285 (15)	0.0115 (13)	0.0054 (12)	0.0066 (12)
C24	0.053 (2)	0.0328 (17)	0.044 (2)	0.0022 (16)	−0.0047 (16)	0.0020 (15)
C36	0.044 (2)	0.060 (2)	0.0438 (19)	0.0244 (18)	0.0135 (16)	0.0187 (17)
C22	0.048 (2)	0.064 (2)	0.043 (2)	0.0007 (19)	−0.0078 (16)	0.0201 (18)
C23	0.081 (3)	0.043 (2)	0.049 (2)	−0.009 (2)	0.023 (2)	0.0077 (17)
C35	0.040 (2)	0.044 (2)	0.061 (2)	−0.0057 (16)	0.0009 (17)	0.0230 (17)
C27	0.069 (3)	0.057 (2)	0.040 (2)	0.006 (2)	−0.0143 (18)	0.0160 (17)
C37	0.070 (3)	0.095 (4)	0.067 (3)	0.025 (3)	0.020 (2)	0.043 (3)
C38	0.095 (4)	0.092 (4)	0.066 (3)	0.043 (3)	0.026 (3)	0.038 (3)
C28	0.091 (5)	0.037 (3)	0.037 (3)	0.015 (3)	−0.010 (3)	0.008 (2)
C29	0.079 (5)	0.059 (4)	0.042 (3)	0.010 (4)	−0.009 (3)	0.020 (3)
C30	0.072 (5)	0.036 (3)	0.074 (5)	0.013 (3)	−0.004 (3)	−0.004 (3)
C31	0.075 (4)	0.042 (3)	0.037 (3)	0.006 (3)	−0.001 (3)	0.007 (2)

### Geometric parameters (Å, º)

**Table:**

O5—C32	1.367 (3)	C7—H7C	0.9600
O5—C33	1.441 (3)	C19—C20	1.388 (4)
O6—C35	1.422 (4)	C19—C18	1.396 (4)
O6—C34	1.424 (4)	C25—C3	1.463 (4)
N2—C13	1.347 (3)	C8—H8A	0.9600
N2—C12	1.349 (3)	C8—H8B	0.9600
N2—C14	1.470 (3)	C8—H8C	0.9600
O8—C18	1.358 (4)	C26—C27	1.479 (5)
O8—C22	1.417 (4)	C26—H26A	0.9700
O4—C32	1.212 (3)	C26—H26B	0.9700
O10—C20	1.365 (4)	C24—H24A	0.9600
O10—C24	1.424 (4)	C24—H24B	0.9600
O2—C25	1.353 (4)	C24—H24C	0.9600
O2—C26	1.440 (4)	C36—C35	1.473 (5)
N1—C2	1.375 (4)	C36—C37	1.534 (5)
N1—C6	1.376 (4)	C36—H36A	0.9700
N1—H1	0.8600	C36—H36B	0.9700
O9—C19	1.373 (4)	C22—H22A	0.9600
O9—C23	1.435 (5)	C22—H22B	0.9600
O1—C25	1.215 (4)	C22—H22C	0.9600
C32—C5	1.460 (4)	C23—H23A	0.9600
O7—C15	1.211 (4)	C23—H23B	0.9600
C10—C9	1.384 (4)	C23—H23C	0.9600
C10—C11	1.386 (4)	C35—H32A	0.9700
C10—H10	0.9300	C35—H32B	0.9700
C13—C9	1.385 (4)	C27—H27A	0.9700
C13—H13	0.9300	C27—H27B	0.9700
C4—C3	1.520 (4)	C37—C38	1.452 (6)
C4—C5	1.528 (4)	C37—H37A	0.9700
C4—C9	1.537 (4)	C37—H37B	0.9700
C4—H4	0.9800	C38—H38A	0.9600
C5—C6	1.356 (4)	C38—H38B	0.9600
C14—C15	1.518 (4)	C38—H38C	0.9600
C14—H14A	0.9700	C28—C29	1.490 (8)
C14—H14B	0.9700	C28—H28A	0.9700
C6—C7	1.498 (4)	C28—H28B	0.9700
C16—C17	1.391 (4)	C29—C30	1.510 (9)
C16—C21	1.399 (4)	C29—H29A	0.9700
C16—C15	1.485 (4)	C29—H29B	0.9700
C12—C11	1.375 (4)	C30—C31	1.501 (9)
C12—H12	0.9300	C30—H30A	0.9700
C11—H11	0.9300	C30—H30B	0.9700
C33—C34	1.489 (4)	C31—H31A	0.9600
C33—H33A	0.9700	C31—H31B	0.9600
C33—H33B	0.9700	C31—H31C	0.9600
C2—C3	1.349 (4)	C28B—C29B	1.544 (17)
C2—C8	1.501 (4)	C28B—H28C	0.9700
C21—C20	1.387 (4)	C28B—H28D	0.9700
C21—H21	0.9300	C29B—C30B	1.520 (16)
O3—C28B	1.404 (15)	C29B—H29C	0.9700
O3—C27	1.417 (4)	C29B—H29D	0.9700
O3—C28	1.427 (6)	C30B—C31B	1.486 (17)
C17—C18	1.390 (4)	C30B—H30C	0.9700
C17—H17	0.9300	C30B—H30D	0.9700
C34—H34A	0.9700	C31B—H31D	0.9600
C34—H34B	0.9700	C31B—H31E	0.9600
C7—H7A	0.9600	C31B—H31F	0.9600
C7—H7B	0.9600		
			
C32—O5—C33	116.7 (2)	C27—C26—H26A	110.1
C35—O6—C34	111.0 (2)	O2—C26—H26B	110.1
C13—N2—C12	121.7 (2)	C27—C26—H26B	110.1
C13—N2—C14	119.6 (2)	H26A—C26—H26B	108.4
C12—N2—C14	118.7 (2)	O8—C18—C17	124.9 (3)
C18—O8—C22	117.6 (3)	O8—C18—C19	115.4 (3)
C20—O10—C24	118.0 (3)	C17—C18—C19	119.8 (3)
C25—O2—C26	114.4 (2)	O10—C24—H24A	109.5
C2—N1—C6	124.0 (2)	O10—C24—H24B	109.5
C2—N1—H1	118.0	H24A—C24—H24B	109.5
C6—N1—H1	118.0	O10—C24—H24C	109.5
C19—O9—C23	111.4 (3)	H24A—C24—H24C	109.5
O4—C32—O5	121.2 (2)	H24B—C24—H24C	109.5
O4—C32—C5	128.1 (3)	C35—C36—C37	111.8 (3)
O5—C32—C5	110.7 (2)	C35—C36—H36A	109.3
C9—C10—C11	120.0 (3)	C37—C36—H36A	109.3
C9—C10—H10	120.0	C35—C36—H36B	109.3
C11—C10—H10	120.0	C37—C36—H36B	109.3
N2—C13—C9	120.7 (2)	H36A—C36—H36B	107.9
N2—C13—H13	119.7	O8—C22—H22A	109.5
C9—C13—H13	119.7	O8—C22—H22B	109.5
C3—C4—C5	111.6 (2)	H22A—C22—H22B	109.5
C3—C4—C9	110.2 (2)	O8—C22—H22C	109.5
C5—C4—C9	108.0 (2)	H22A—C22—H22C	109.5
C3—C4—H4	109.0	H22B—C22—H22C	109.5
C5—C4—H4	109.0	O9—C23—H23A	109.5
C9—C4—H4	109.0	O9—C23—H23B	109.5
C6—C5—C32	120.9 (2)	H23A—C23—H23B	109.5
C6—C5—C4	120.8 (2)	O9—C23—H23C	109.5
C32—C5—C4	118.0 (2)	H23A—C23—H23C	109.5
N2—C14—C15	110.2 (2)	H23B—C23—H23C	109.5
N2—C14—H14A	109.6	O6—C35—C36	112.1 (3)
C15—C14—H14A	109.6	O6—C35—H32A	109.2
N2—C14—H14B	109.6	C36—C35—H32A	109.2
C15—C14—H14B	109.6	O6—C35—H32B	109.2
H14A—C14—H14B	108.1	C36—C35—H32B	109.2
C5—C6—N1	119.6 (3)	H32A—C35—H32B	107.9
C5—C6—C7	126.7 (3)	O3—C27—C26	105.7 (3)
N1—C6—C7	113.7 (2)	O3—C27—H27A	110.6
C17—C16—C21	121.1 (3)	C26—C27—H27A	110.6
C17—C16—C15	117.7 (3)	O3—C27—H27B	110.6
C21—C16—C15	121.2 (3)	C26—C27—H27B	110.6
N2—C12—C11	119.4 (2)	H27A—C27—H27B	108.7
N2—C12—H12	120.3	C38—C37—C36	115.5 (4)
C11—C12—H12	120.3	C38—C37—H37A	108.4
C12—C11—C10	119.9 (3)	C36—C37—H37A	108.4
C12—C11—H11	120.1	C38—C37—H37B	108.4
C10—C11—H11	120.1	C36—C37—H37B	108.4
O7—C15—C16	122.1 (3)	H37A—C37—H37B	107.5
O7—C15—C14	120.1 (3)	C37—C38—H38A	109.5
C16—C15—C14	117.7 (2)	C37—C38—H38B	109.5
C10—C9—C13	118.3 (2)	H38A—C38—H38B	109.5
C10—C9—C4	121.3 (2)	C37—C38—H38C	109.5
C13—C9—C4	120.4 (2)	H38A—C38—H38C	109.5
O5—C33—C34	108.9 (2)	H38B—C38—H38C	109.5
O5—C33—H33A	109.9	O3—C28—C29	113.3 (5)
C34—C33—H33A	109.9	O3—C28—H28A	108.9
O5—C33—H33B	109.9	C29—C28—H28A	108.9
C34—C33—H33B	109.9	O3—C28—H28B	108.9
H33A—C33—H33B	108.3	C29—C28—H28B	108.9
C3—C2—N1	120.0 (2)	H28A—C28—H28B	107.7
C3—C2—C8	126.3 (3)	C28—C29—C30	115.8 (6)
N1—C2—C8	113.7 (3)	C28—C29—H29A	108.3
C20—C21—C16	118.9 (3)	C30—C29—H29A	108.3
C20—C21—H21	120.5	C28—C29—H29B	108.3
C16—C21—H21	120.5	C30—C29—H29B	108.3
C28B—O3—C27	119.8 (8)	H29A—C29—H29B	107.4
C28B—O3—C28	30.1 (8)	C31—C30—C29	112.9 (6)
C27—O3—C28	113.2 (3)	C31—C30—H30A	109.0
C18—C17—C16	119.4 (3)	C29—C30—H30A	109.0
C18—C17—H17	120.3	C31—C30—H30B	109.0
C16—C17—H17	120.3	C29—C30—H30B	109.0
O6—C34—C33	110.7 (2)	H30A—C30—H30B	107.8
O6—C34—H34A	109.5	C30—C31—H31A	109.5
C33—C34—H34A	109.5	C30—C31—H31B	109.5
O6—C34—H34B	109.5	H31A—C31—H31B	109.5
C33—C34—H34B	109.5	C30—C31—H31C	109.5
H34A—C34—H34B	108.1	H31A—C31—H31C	109.5
C6—C7—H7A	109.5	H31B—C31—H31C	109.5
C6—C7—H7B	109.5	O3—C28B—C29B	101.9 (12)
H7A—C7—H7B	109.5	O3—C28B—H28C	111.4
C6—C7—H7C	109.5	C29B—C28B—H28C	111.4
H7A—C7—H7C	109.5	O3—C28B—H28D	111.4
H7B—C7—H7C	109.5	C29B—C28B—H28D	111.4
O9—C19—C20	120.4 (3)	H28C—C28B—H28D	109.3
O9—C19—C18	119.3 (3)	C30B—C29B—C28B	110.1 (13)
C20—C19—C18	120.3 (3)	C30B—C29B—H29C	109.6
O1—C25—O2	120.9 (3)	C28B—C29B—H29C	109.6
O1—C25—C3	127.7 (3)	C30B—C29B—H29D	109.6
O2—C25—C3	111.4 (2)	C28B—C29B—H29D	109.6
O10—C20—C21	124.7 (3)	H29C—C29B—H29D	108.2
O10—C20—C19	114.8 (3)	C31B—C30B—C29B	112.6 (17)
C21—C20—C19	120.5 (3)	C31B—C30B—H30C	109.1
C2—C3—C25	121.1 (3)	C29B—C30B—H30C	109.1
C2—C3—C4	121.0 (2)	C31B—C30B—H30D	109.1
C25—C3—C4	117.8 (2)	C29B—C30B—H30D	109.1
C2—C8—H8A	109.5	H30C—C30B—H30D	107.8
C2—C8—H8B	109.5	C30B—C31B—H31D	109.5
H8A—C8—H8B	109.5	C30B—C31B—H31E	109.5
C2—C8—H8C	109.5	H31D—C31B—H31E	109.5
H8A—C8—H8C	109.5	C30B—C31B—H31F	109.5
H8B—C8—H8C	109.5	H31D—C31B—H31F	109.5
O2—C26—C27	107.9 (3)	H31E—C31B—H31F	109.5
O2—C26—H26A	110.1		

### Hydrogen-bond geometry (Å, º)

**Table:**

*D*—H···*A*	*D*—H	H···*A*	*D*···*A*	*D*—H···*A*
N1—H1···Br1^i^	0.86	2.61	3.421 (2)	157

Symmetry code: (i) *x*, *y*+1, *z*.
